# Potential of Thirteen Urban Greening Plants to Capture Particulate Matter on Leaf Surfaces across Three Levels of Ambient Atmospheric Pollution

**DOI:** 10.3390/ijerph16030402

**Published:** 2019-01-31

**Authors:** Yanmei Li, Shaojun Wang, Qibo Chen

**Affiliations:** College of Ecology and Soil & Water Conservation, Southwest Forestry University, 300 Bailongsi, Kunming 650224, China; kmlymei@126.com (Y.L.); chengqb@swfu.edu.cn (Q.C.)

**Keywords:** air particulate matter, functional zone, micromorphological traits, trees and shrubs

## Abstract

The potential of urban greening plants to capture particulate matter (PM) from the ambient atmosphere is contingent on interactions between the level of pollution and leaf surfaces. For this study, thirteen plant species were investigated to quantify their capacity of PM accumulation under three atmospheric environments, that is, industrial, traffic and university campus (control), in Kunming City (Southwest China). The sampled sites represented different pollution levels (that is, high pollution, slight pollution and clean air, respectively). The plant species differed in their accumulation of PM by six- to eight-fold across the three sites. *Magnolia grandiflora* was the most efficient evergreen tree species, whereas *Platanus* *acerifolia* had the highest capture of PM among deciduous trees. The accumulation capacity of the same species varied with the degree of pollution. For example, *Osmanthus fragrans*, *Loropetalum chinense* and *Cinnamomum japonicum* were highly efficient for the capture of PM in the traffic and university campus areas; however, they exhibited medium accumulation in the industrial area. *Prunus majestica* demonstrated an intermediate accumulation capacity in the industrial area, but was low in the traffic and university campus areas. The capturing capacity of the same genus was also different among the different levels of pollution. For example, *C. japonicum* had a 2.9–4.2-times higher PM accumulation than did *C. camphora* across the three sites. There were significant differences in leaf surface area, stomata density/length, guard cell area, and trichome density/length among these species. The species-specific efficacy of PM capture was primarily contributed to by leaf size and surface roughness, stomata density, and trichome length. In particular, hairy-leaf leaves with medium stomatal density exhibited higher PM capture. Therefore, leaf micromorphology, leaf size and longevity appeared to be significant predictive factors for the accumulation of PM, which may aid in the selection of greening plant species for the remediation of pollutants in urban areas.

## 1. Introduction 

Particulate matter (PM) in the ambient atmosphere serves as an important indicator for the evaluation of urban air quality [1,2]. Atmospheric PM originates primarily from the anthropogenic discharges of vehicles, coal burning, industrial production, domestic pollution and fertilizer production [3,4]. PM is a mixture of hazardous substances and other suspended matter in the atmosphere, which can exert serious negative health effects on urban populations that typically manifest as degradative respiratory and cardiovascular diseases [5,6]. 

Urban greening plants have the potential to purify contaminated air through the capture of PM on the surfaces of their leaves [7,8]. They reduce the concentrations of fine particulate atmospheric pollutants, thus lowering the risks of disease for urban inhabitants [9,10]. The extensive surface areas (per unit ground area) of the leaves of urban plants have higher efficiencies for the capture of airborne PM, in contrast to other surfaces in these environments [11,12]. Urban plants have been reported to reduce 9.1% of PM concentrations in Shanghai, China, compared with external woodlands [13]. The PM captured by urban plants may also remove heavy metal-associated PM from the ambient atmosphere. 

Various plant species differ in terms of their capacity for PM accumulation from the ambient atmosphere. Trees have improved efficiencies for the capture of PM due to their higher leaf surface areas, compared with shrubs and herbs [14]. Trees are often large with solid structures, which can generate air turbulence to increase the accumulation of PM on their leaf surfaces [15,16]. Broadleaved species exhibit higher PM capture capacities than do those with smoother blade-like surfaces [17]. Furthermore, evergreen broadleaved species have important implications for air purification, as they can continuously accumulate PM. 

At the single-leaf scale, various plant species have different PM purification capacities, as they develop distinct leaf dimensions and morphological traits (that is, ravine, stomata and epidermal trichome) that effectively capture PM from the atmosphere [18,19]. The dimensions of leaves may have significant effects on the accumulation of PM, where complex leaf shapes (e.g., lobed leaves) show greater potential for the capture of PM than those that are simple [20]. Leaf surfaces with trichomes, ridges and epicuticular waxes can enhance air filtration processes in contrast to smooth surfaces [20,21]. Higher levels of leaf stomata have also been reported to have potential for the capture of ultrafine PM on leaf surfaces [8]. 

Greening plants may serve as a cost effective and environmentally compatible means for the biomonitoring of pollutants in urban settings, as plant materials (e.g., tree leaves and bark) are easy to collect, may be analyzed cheaply, and produce no secondary pollution [22,23]. Thus, plants can be widely utilized for the removal of airborne contaminants in urban environments. However, little is known in regard to the differences in the efficacy of PM capture among various species of urban greening plants. The ability to capture PM is an important trait in the selection of optimal plant species for urban greening [24]. Therefore, it is critical to identify the capacity of different plant species to filter air-borne PM pollutants, toward optimizing the benefits of these plants in various urban environments [25]. 

In recent decades, atmospheric PM has increased in conjunction with the advancement of urbanization, industrialization and rural–urban migration in Kunming [19], which can exert severe negative health effects on its residents [19]. Therefore, a number of plant species are being employed in Kunming City for the purification of air pollutants. It is crucial to identify the differences between these species for their PM capturing capacity, to subsequently select the optimal plant species for this application. We examined the PM sequestering abilities of 13 common greening plants in three functional zones under different levels of pollution in Kunming City. Therefore, the main questions were: 1) did these plant species differ in their ability to capture PM on their leaf surfaces? and 2) did the surface capture of PM vary with leaf traits (e.g., size, longevity, and surface micromorphological structures such as roughness, stomata density and trichome density)?

## 2. Materials and Methods

### 2.1. Site Design

**Figure 1:**
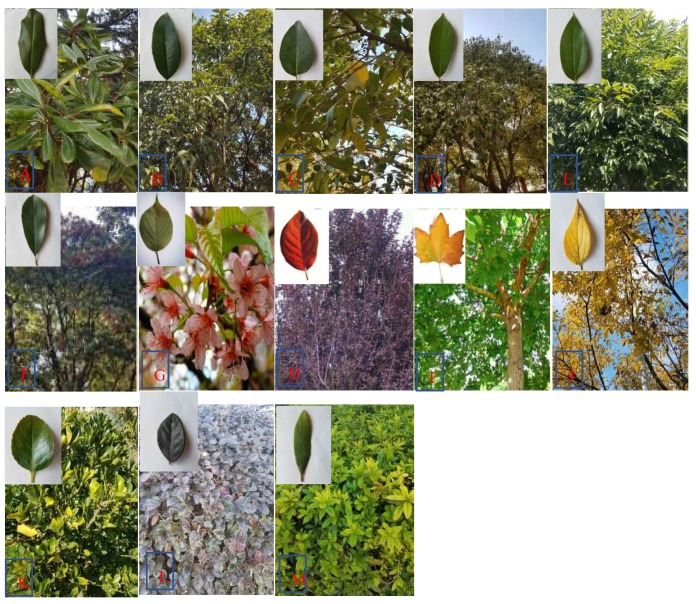
Pictures of the selected thirteen plant species in Kunming City. A: *Magnolia grandiflora* Linn; B: *Osmanthus fragrans* (Thunb.) Lour.; C: *Ligustrun lucidum* Ait; D: *Cinnamomum camphora* (L.) Presl.; E: *Cinnamomum japonicum* Sieb; F: *Photinia glomerata* Rehd. et Wils; G: *Prunus majestica* Koehne; H: *Prunus cerasifera* f. atropurpurea; I: *Platanus acerifolia* Ait.; J: *Celtis kunmingensis* Cheng et Hong; K: *Euonymus japonica* Thunb.; L: *Loropetalum chinense* var. rubrum; M: *Rhododendron pulchrum* Sweet.

**Figure 2:**
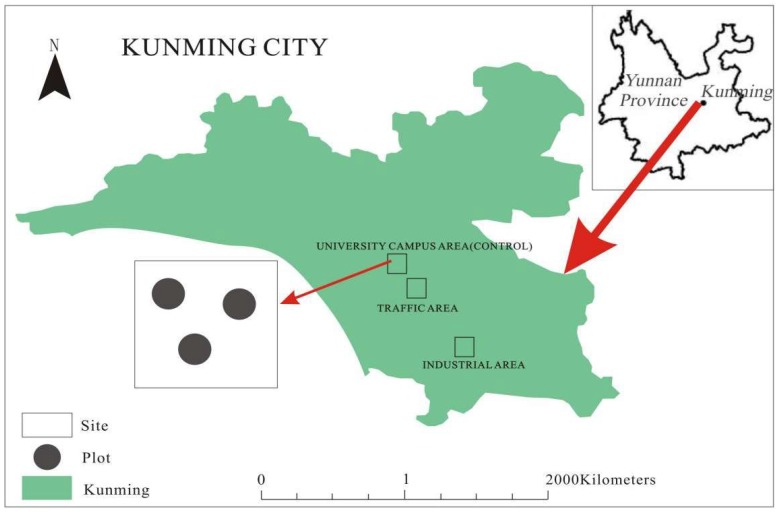
Site arrangement in this study.

The thirteen greening plants (at the middle-age growth stage) (Figure 1 and Table 1) selected for this study were common species widely cultivated across three functional zones, that is, industrial, traffic and university campus (control), in Kunming City, Southwest China. Five similar individuals per plant species were employed as replicates. These plants were free of pollution with little, or no, disease or pests prior to planting. These sites (each with three repetitions) (Figure 2) were considered as being under different contamination levels (that is, high pollution, slight pollution and clean) (Table 2). These plant species were randomized in approximately the same manner in fields with minimal meteorological variations in site conditions (e.g., wind and precipitation). Thus, we could examine if certain species had higher or lower PM accumulation levels at different pollution-level sites. The PM concentrations were measured at 5 min intervals using a Laser Dust Monitor (GZ-5, 10% precision, Shanghai Gaozhi Precision Instrument Co., Ltd., Shanghai, China), whereas wind speed and direction were determined at 2 min intervals by a hand-held Electronic Anemorumbometer (FC-16025, Shanghai Precision Instrument Co., Ltd., Shanghai, China). 

### 2.2. Sample Collection and PM Analysis

Plant leaves (300 and 400 cm^2^ per species) were sampled from the traffic side of 13 plants at a 0.6–2.0 m height aboveground, depending on the plant structure [26]. The samples were collected into labelled paper bags, and then kept in clean storage boxes under room temperature in the laboratory prior to measurements [26]. Two samples from two growth seasons (June and September 2016) were collected at the same locations on the same day. Generally, precipitation of more than 15 mm washed the dust off the leaves; hence, the plants began a new dust release/retention cycle [27]. Leaf sampling began in June, five days after a period of heavy rain (19 mm), which ensured the same initial PM concentrations on the leaf surfaces. 

The nanoporous (0.45 μm in diameter) materials used for filtering were weighed after 30 min of drying under 60 ºC in a drying apparatus, with stabilized humidity in a weighing room. The filters were preweighed on a XS105DU balance (Mettler-Toledo International Inc., Zurich, Switzerland). The PM was rinsed off through agitation for 60 s in a glass container with 250 mL water. The liquid was subsequently vacuum-filtered through the filters, which were then weighed after drying. The concentrations of captured PM were then calculated for each sample. The total leaf areas were determined with a leaf area meter (Licor LI-3050A/4, LICOR, Inc., Lincoln, NE, USA) and Skye Leaf software (Wanshen testing technology co. Ltd., China). The PM concentrations were then calculated per g m^−2^ of leaf area. 

The leaves of selected species (see above) were used to correlate the PM capture to leaf traits, and the surface area per blade and leaf longevity (deciduous or evergreen) were recorded. The micromorphological structures (that is, ravine, stomata and epidermal trichome) were subsequently scanned using an E-1010 scanning electron microscope (SEM, Hitachi, Japan). Four tissue blocks (5 × 5 mm) per lamina (five leaves per plant species) were sampled and fixed with a 2.5% glutaraldehyde solution, cleaned with a 0.1 M phosphate buffer solution, dehydrated with gradient-ethanol dehydration, fixed with *n*-butyl alcohol, and coated via E-1010 ion-plating (COXEM Co., Ltd., Daejeon, South Korea). The leaf roughness was evaluated by a experiential scale (1 = low and 5 = high) under a microscope (10x magnification). The stomatal density was observed under 300x magnification, while the numbers and lengths of surface trichomes were observed under 100x magnification.

### 2.3. Statistical Analyses 

All data were verified for normal distributions and variance homogeneities prior to analysis. Multiple comparisons of means between species were performed using the Duncan test. A least significant difference (LSD) test was used to compare the means at a *p* < 0.05 significance level. The plant species were grouped as three types (that is, low, intermediate or high capacity for PM accumulation), using K-means clustering. The standardization for average PM capture was employed to run the cluster for each site. Multiple regression models were applied to determine the relationships between leaf characteristics and PM deposition. These statistical analyses were conducted with SPSS 22.0 (SPSS for Windows, Chicago, IL, USA). 

## 3. Results 

### 3.1. PM Accumulation among Species and Locations

**Figure 3:**
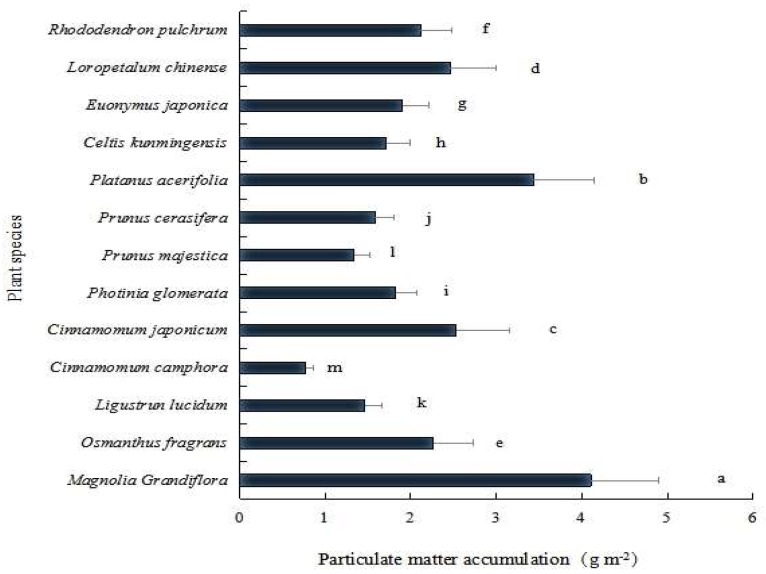
Accumulation of total particulate matter (PM) on the leaf surfaces of tree and shrub species in Kunming City, China. Bars are mean ± SE (standard error). Treatments with the same letter were not significantly different (ANOVA with Duncan test, *p* < 0.05).

The thirteen plant species differed in their capacities for PM capture on their leaf surfaces in Kunming City, which were calculated as the mean values of total PM at three functional zones (that is, industrial, traffic and university campus) (*p* < 0.01; Figure 3). The PM capturing abilities of these plant species were ranked as *Magnolia grandiflora* (4.20 g m^−2^) > *Platanus acerifolia* (3.43 g m^−2^) > *Cinnamomum japonicum* (2.53 g m^−2^) > *Loropetalum chinense* (2.46 g m^−2^) > *Osmanthus fragrans* (2.25 g m^−2^) > *Rhododendron pulchrum* (2.12 g m^−2^) > *Euonymus japonica* (1.9 g m^−2^) > *Photinia glomerata* (1.83 g m^−2^) > *Celtis kunmingensis* (1.71 g m^−2^) > *Prunus cerasifera* (1.6 g m^−2^) > *Ligustrun lucidum* (1.47 g m^−2^) > *Prunus majestica* (1.34 g m^−2^) > *Cinnamomum camphora* (0.99 g m^−2^) (*p* < 0.05; Figure 3).

**Figure 4:**
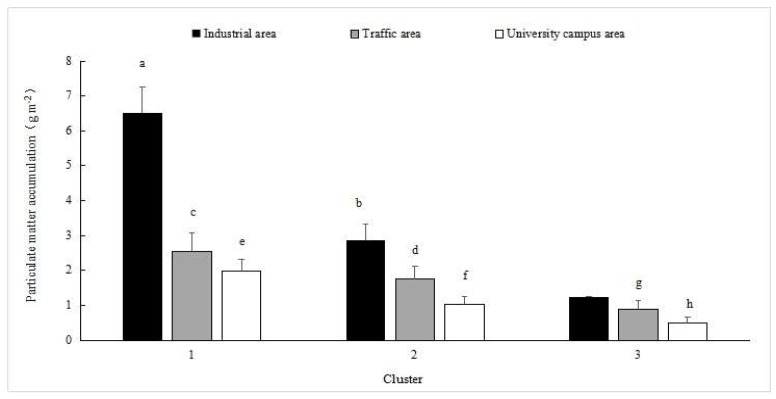
Accumulation of total particulate matter (means ± SE) on the leaves in the three clusters found in the three functional zones. Bars are means ± SE. Treatments with the same letter (a–h) were not significantly different (ANOVA with Duncan test, *p* < 0.05). SE is standard error.

The total PM capture capacities of three sample groups (that is, clusters 1, 2 and 3) varied across the three functional zones (*p* < 0.01; Figure 4). The quantities of PM deposited on the leaf surfaces of the three groups were higher for the industrial area (1.24–7.8 g m^−2^) than for the traffic area (0.87–4.05 g m^−2^) and university campus area (0.4–2.76 g m^−2^), which reflected the effects of different pollutant levels on the PM accumulation at the three sites. The plant species differed in their abilities for PM capture according to clustering. *C*. *camphora* was in the low cluster at the three sites, whereas the *C*. *kunmingensis* and *E*. *japonica* species were in the intermediate cluster, and *M*. *grandiflora* was in the highest cluster. The same species differed in their PM capture abilities at different sites. *O*. *fragrans*, *L*. *chinense* and *C*. *japonicum* were in the cluster with high accumulation for traffic and university campus areas, while they were in the medium cluster for the industrial area. *P*. *majestica* was in the cluster with intermediate accumulation for the industrial area, but was in the low cluster for the traffic and university campus areas. Furthermore, differences among species were also observed within the same genus. *C. camphora* was in the low cluster for all three sites, while *C. japonicum* was in the medium or high cluster for the traffic and university campus areas. For the traffic area, *P. majestica* had low PM capture, whereas *P. cerasifera* was in the cluster with medium accumulation.

**Figure 5:**
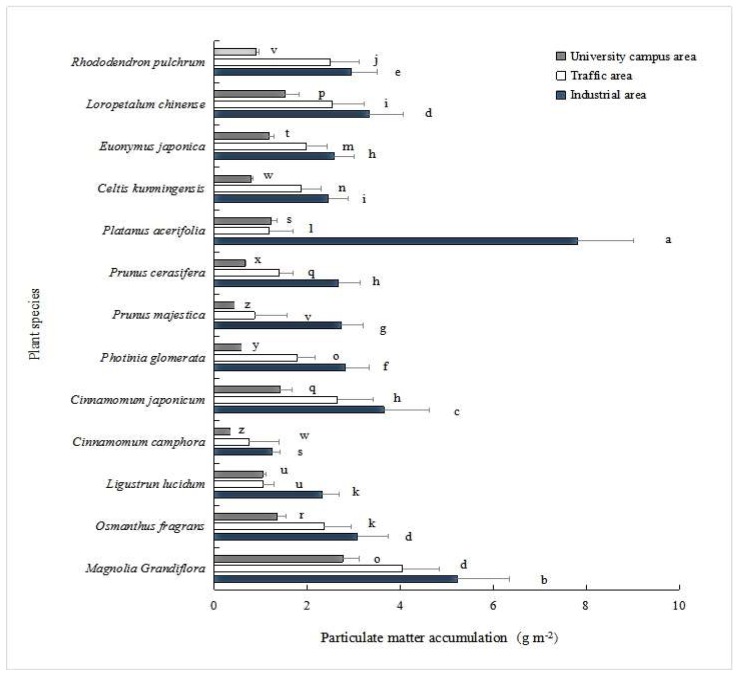
Particulate matter accumulated on leaf surfaces of trees and shrub species in three functional zones (industrial, traffic and university campus) in Kunming City, China. Bars are means ± SE. Treatments with the same letter were not significantly different (ANOVA with Duncan test, *p* < 0.05).

PM capture abilities of these species significantly differed with sites (*p* < 0.05; Figure 5). In the industrial area, *M. grandiflora* and *P. acerifolia* showed high PM accumulation on leaf surfaces (5.22–7.8 g m^−2^) (Figure 5). The *O. fragrans*, *C. japonicum*, *L. chinense*, *R. pulchrum*, *P. majestica*, L. *lucidum*, *P. cerasifera*, *C. kunmingensis*, *P. glomerata* and *E. japonica* species had intermediate PM accumulation (2.31–3.65 g m^−2^), while *C. camphora* contributed considerably lower PM accumulation (1.24 g m^−2^), and was the only species in this cluster. In the traffic area, the species with the largest PM accumulation (2.18–4.05 g m^−2^) included *M. grandiflora*, *C. japonicum*, L. *chinense, R*. *pulchrum*, *O. fragrans* and *P. acerifolia* (Figure 5). The *E. japonica*, *C. kunmingensis*, *P. glomerata* and *P. cerasifera* species accumulated a medium total PM (1.4–1.97 g m^−2^), whereas *L. lucidum*, *P. majestica* and *C. camphora* had lower PM accumulation (0.74–1.04 g m^−2^). Data from the university campus area revealed that *M. grandiflora*, *L. chinense*, *C. japonicum* and *O. fragrans* had the highest PM accumulation (1.36–2.76 g m^−2^) (Table 1). The species with intermediate PM accumulation (0.8–1.23 g m^−2^) were *P. acerifolia*, *E. japonica*, *L. lucidum*, *R. pulchrum* and *C. kunmingensis*. The lowest PM accumulations (0.33–0.66 g m^−2^) were observed on the leaf surfaces of *P. cerasifera, P. glomerata, P. majestica* and *C*. *camphora*. 

### 3.2. Association of Leaf Surface Traits with PM Capture

There were significant differences in leaf areas per leaf, stomata density/length, guard cell area, and trichome density/length among these species (*p* < 0.05; Table 3). Leaf surface traits were closely associated with PM accumulation (Table 3 and Table 4). *M. grandiflora* with the highest PM accumulation had the greatest leaf area per leaf, trichome density/length, and guard cell area, though the leaf surface was relatively smooth with medium stomata density at the lower epidermis. However, *C. camphora* with the lowest PM accumulation had the smoothest leaf surface, and no distribution of trichome, though there was medium stomata density at the lower epidermis. Leaf surface roughness, leaf area per leaf, stomata density and trichome length were important predictors for PM accumulation (Table 4). An increase in leaf surface roughness, leaf area per leaf and trichome length predicted an increase in PM deposition, while an increase in stomata number induced a decrease in PM accumulation. Changes in the leaf micromorphology index revealed a stronger explanation (*R^2^* = 0.992) for the PM accumulation (Table 4).

## 4. Discussion

In the present study, the average PM accumulation (2.12 g m^−2^) of thirteen plant species was similar to the results (2.23 g m^−2^) reported in Guangzhou, in Southern China [27], while it was greater than that (1.69 g m^−2^) in Xi’an, in Northwestern China [28]. This indicated that regionally climatic conditions may be an important factor that affects dust retention on plant leaves. All tested greening plant species differed in the accumulation of PM on their leaves from surrounding atmospheric environments. Generally, tall tree species (e.g., arbor) had a higher potential for the capture of PM on their leaf surfaces. For example, *M*. *grandiflora* and *P*. *acerifolia* accumulated higher quantities of PM on their leaves in contrast to other shorter plant species. Thus, these tall trees may be the most efficient plant species, due to their larger total leaf areas and sizes, and more complex tree crown structures, which facilitate the capture of PM on their leaves [14,15]. However, the tall trees of *C*. *camphora* had the lowest PM accumulation, indicating that tree height is not the only factor determining the abilities for PM accumulation. The shrubs, such as *L*. *chinense* and *R*. *pulchrum,* had medium PM accumulation. This may have been attributed to the easy exposure to soil splash on the leaves of low-height plant species compared with tall trees. These low-growing shrubs often had higher PM capture efficiencies compared with other tree species [26]. Plant height had no significant impact on the accumulation of PM [29,30]. Further, several studies revealed that evergreen species had higher PM capture than did deciduous species [24]. Although there was no difference in the effect of leaf longevity on PM accumulation, evergreen species that grew year-round may have possessed a greater temporal advantage, which facilitated the higher accumulation of PM over deciduous species. 

Some plant species of the same genus had different PM capturing capacities. For example, *P. majestica* showed low PM capture (0.42 and 0.87 g m^−2^, respectively) in the traffic and campus areas; however, *P. cerasifera* accumulated medium quantities of PM (0.67 and 1.4g m^−2^, respectively) at the same sites. The reason may have been differences in surface traits among these *Pinus* species. On the other hand, site conditions may have affected the PM capture of the same genus species. *C. camphora* showed low efficiency in PM capture at all three sites, while *C. japonicum* was at the medium or high level of PM accumulation in the traffic and university campus areas. Some species accumulated higher PM at sites with higher pollution levels but had lower efficiencies in cleaner environments. This might have been related to different PM concentrations, or fractions, at different sites. Thus, different capacities for the capture of PM on leaves may be attributed not only to plant species, but also site environmental factors [20]. The accumulation abilities of certain plant species had a close association with the deposition rate and level of contamination, which were associated with specific site environments [7,9]. The test species at the three locations under different pollution conditions provided data on which plants in the same cluster had higher or lower PM deposition. Some greening plant species had the highest (e.g., *M*. *grandiflora*) or lowest (e.g., *C*. *camphora*) accumulation of PM at relatively high levels of contamination, while they also had a higher or lower capacity for PM capture at sites with low-level contamination. The same species (e.g., *P*. *acerifolia*) accumulated higher PM at sites with higher levels of pollution, but had lower efficiencies in cleaner environments. Furthermore, certain species (e.g., *O*. *fragrans*, *L*. *chinense* and *C*. *japonicum*) had high capacities for PM accumulation in relatively highly polluted areas, but accumulated less PM in cleaner areas. Therefore, the same genus (or species) that differed in the accumulation of PM on their leaf surfaces might have been dependent on specific leaf traits and contamination levels.

Several studies have suggested that the capacities of greening plants to capture PM was principally determined by the morphological structures of leaves (e.g., cuticle roughness, stomata densities, morphologies and quantities of epidermal trichomes), leaf areas, as well as prevailing meteorological conditions [28,29]. For this study, the leaf size, surface roughness, and stomata density and length were important predictors for the accumulation of PM. Increased leaf size and roughness predicted higher PM accumulation, while increased stomata density and length indicated a decrease in PM capture. *M. grandiflora*, which is a species with relatively smooth leaf surfaces, had the highest quantity of PM retention, which may have been attributed to a higher leaf area per leaf, and trichome density and length, with average stomata density. Research has also revealed that leaves with low stomatal density, high stomatal conductance and greater leaf hairiness had a higher PM capture capacity [31]. Misson et al. (2005) [32] and Enbao et al. (2017) [33] reported that high stomatal density suppressed the dust retention of plants, through the formation of strong hydrophobicity, which reduced the affinity and contact area between blades and particulate pollutants. 

PM was typically captured on the adaxial leaf surfaces, while only smaller PM fractions accumulated on the abaxial surfaces of stomatal areas. A number of studies have observed that a small portion of PM was also captured on the undersides of leaves [34], which indicated that high stomatal densities may not be necessary to induce high PM accumulation. For our study, plant species (e.g., *M. grandiflora*, *P*. *acerifolia* and *C. japonicum*) with high PM accumulations had a medium stomatal density of 32–48 ind. under 300x magnification. These results were similar to those reported by Liu et al. [27]. Thus, stomatal density may have a threshold effect on the accumulation of PM. Furthermore, the hairy undersides of leaves had a higher capacity for PM capture than hairless upper sides [17]. We surmised that hairy leaves with medium stomatal densities may exhibit higher PM capture capacities. 

Trees in this study had a more highly efficient capacity for the capture of PM pollution, while shrubs had medium PM accumulation capacities. Smaller shrub plants may be an important choice for the lower strata of urban greening [35]. However, currently, the contributions of shrubs to PM capture are often neglected. Thus, both shrub and tree species should be considered in the design of urban greening for the mitigation of urban atmospheric pollution. 

## 5. Conclusions

The present study documented the different PM capture abilities of 13 urban greening plant species, which were selected to reduce human exposure to air pollution. We found that the distinct efficiency of the same genus or species in PM accumulation varied with the level of pollution. Furthermore, leaf size, longevity and micromorphological structures had crucial roles in the determination of PM capture. These data may be critical for the evaluation of the benefits of urban plants on PM capture. Therefore, the identification and quantification of the efficacy of these plant species, and their regulating factors, may be significant toward the selection of greening plant species for pollution control.

## Figures and Tables

**Table 1:** Plant species used and clustering analysis with the surface accumulation of particulate matter (PM) as variables.

Species	Family	Cluster in Industrial Area	Cluster in Traffic Area	Cluster in Campus Area
*Magnolia grandiflora* Linn	*Magnoliaceae*	1	1	1
*Osmanthus fragrans* (Thunb.) Lour.	*Oleaceae*	2	1	1
*Ligustrun lucidum* Ait	*Oleaceae*	2	3	2
*Cinnamomum camphora* (L.) Presl.	*Lauraceae*	3	3	3
*Cinnamomum japonicum* Sieb	*Lauraceae*	2	1	1
*Photinia glomerata* Rehd. et Wils.	*Rosaceae*	2	2	3
*Prunus majestica* Koehne	*Rosaceae*	2	3	3
*Prunus cerasifera* f. atropurpurea	*Rosaceae*	2	2	3
*Platanus acerifolia* Ait.	*Platanaceae*	1	2	2
*Celtis kunmingensis* Cheng et Hong	*Ulmaceae*	2	2	2
*Euonymus japonica* Thunb.	*Celastraceae*	2	2	2
*Loropetalum chinense* var. rubrum	*hamamelidaceae*	2	1	1
*Rhododendron pulchrum* Sweet	*Ericaceae*	2	1	2

PM concentrations of cluster 1 in industrial, traffic and campus areas were >5.22 g m^−2^, >2.35 g m^−2^ and >1.4 g m^−2^, respectively; those of cluster 2 were 1.24–5.22 g m^−2^, 1.05–2.35 g m^−2^ and 0.67–1.4 g m^−2^, respectively; those of cluster 3 were <1.24 g m^−2^, <1.05 g m^−2^ and <0.67 g m^−2^, respectively.

**Table 2:** Environmental conditions across the three functional zones (industrial, traffic and university campus) in Kunming City, Southwest China.

Site	Geographic Position	Level of Air Pollution	Initial Air PM Concentration (mg m^−3^)	Annual Mean Air Temperature (°C)	Annual Mean Precipitation (mm)	Wind Speed during the Study (m/s)	Wind Direction during the Study
Industrial area	25°6′N, 102°8’E	Higher industrial pollution	488.07 ± 57.52	14.9	1000.5	3.02 ± 0.41	Southwest
Traffic area	25°21′N, 102°30’E	Slight traffic pollution	87.41 ± 6.36	14.5	1031	2.36 ± 0.22	Southwest
University campus area (Control site)	25°18′N, 102°27’E	Clean air	39.1 ± 2.45	14.3	1035	2.17 ± 0.27	Southwest

**Table 3:** Leaf surface traits of 13 trees and shrub species in Kunming City, China.

Species	Leaf Longevity	LA (cm^2^ leaf^−1^)	R	RBA (μm)	RBL (µm)	SD (300× view^−^^1^)	SL (µm)	SB (µm)	GC (µm²)	TN (300× view^−^^1^)	TL (µm)
*grandiflora*	Evergreen	58.27 ± 9.6 ^a^	1	-	2.12 ± 0.41 ^e^	37 ± 7 ^h^	6.8 ± 0.74 ^h^	2.46 ± 0.55 ^c^	625.5 ± 102 ^a^	73 ± 10 ^a^	168 ± 19 ^c^
*O*. *fragrans*	Evergreen	39.82 ± 9 ^b^	4	3.61 ± 0.23 ^e^	2.03 ± 0.32 ^e^	113 ± 35 ^a^	9.43 ± 0.41 ^e^	3.38 ± 0.41 ^b^	391.2 ± 64 ^d^	-	-
*L*. *lucidum*	Evergreen	16.72 ± 7.4 ^g^	2	3.09 ± 0.18 ^e^	4.01 ± 0.15 ^c^	30 ± 7 ^k^	16.48 ± 3.74 ^a^	6.26 ± 0.32 ^a^	618.7 ± 96 ^b^	-	-
*C*. *camphora*	Evergreen	20.57 ± 2.9 ^e^	1	8.21 ± 1.08 ^b^	5.04 ± 0.66 ^b^	48 ± 8 ^f^	12.85 ± 2.47 ^b^	3.32 ± 0.78 ^b^	171.3 ± 51 ^g^	-	-
*C*. *japonicum*	Evergreen	21.23 ± 9 ^e^	5	1.44 ± 1.08 ^f^	2.96 ± 0.66 ^e^	25 ± 5 ^l^	11.53 ± 2.32 ^c^	1.98 ± 0.36 ^d^	93.5 ± 46.5 ^i^	12 ± 9 ^d^	81.25 ± 9 ^f^
*P*. *glomerata*	Evergreen	30.16 ± 6.3 ^d^	3	5.32 ± 0.48 ^c^	1.9 ± 0.34 ^f^	57 ± 10 ^e^	4.77 ± 0.57 ^k^	1.75 ± 0.25 ^d^	85.8 ± 21 ^j^	-	-
*P*. *majestica*	Deciduous	17.81 ± 1.8 ^f^	2	2.28 ± 0.17 ^f^	5.39 ± 0.75 ^b^	63 ± 14 ^d^	8.78 ± 1.34 ^f^	2.41 ± 0.51 ^c^	204.1 ± 34 ^f^	9 ± 7 ^d^	213 ± 20 ^b^
*P*. *cerasifera*	Deciduous	15.32 ± 1.6 ^h^	2	4.05 ± 0.64 ^d^	2.22 ± 0.48 ^c^	73 ± 12 ^c^	4.14 ± 0.42 ^l^	2.59 ± 0.58 ^c^	261.7 ± 68 ^e^	-	-
*P*. *acerifolia*	Deciduous	34.64 ± 6.9 ^c^	5	4.88 ± 0.51 ^d^	4.05 ± 0.55 ^c^	32 ± 8 ^j^	10.8 ± 2.26 ^d^	3.86 ± 0.91 ^b^	529.9 ± 95 ^c^	68 ± 12 ^b^	42.3 ± 9 ^g^
*C*. *kunmingensis*	Deciduous	14.47 ± 1.4 ^i^	3	-	2.06 ± 0.34 ^e^	101 ± 22 ^b^	6.2 ± 0.84 ^i^	0.8 ± 0.18 ^e^	138.3 ± 45 ^h^	3 ± 4 ^e^	137 ± 17 ^d^
*E*. *japonica*	Evergreen	0.86 ± 0.9 ^k^	4	3.3 ± 0.26 ^e^	5.53 ± 0.69 ^b^	43 ± 7 ^g^	7.69 ± 0.96 ^g^	3.58 ± 0.88 ^b^	42.15 ± 11 ^l^	-	-
*L*. *chinense*	Evergreen	9.31±1.1 ^j^	5	9.28 ± 1.34 ^a^	5.85 ± 0.87 ^b^	34 ± 6 ^i^	8.18 ± 1.08 ^g^	3.48 ± 0.87 ^b^	210.9 ± 46 ^c^	30 ± 8 ^c^	100 ± 15 ^e^
*R*. *pulchrum*	Evergreen	7.88 ± 0.8 ^l^	4	5.08 ± 0.96 ^c^	6.51 ± 1.16 ^a^	61 ± 16 ^b^	5.59 ± 0.66 ^j^	2.42 ± 0.52 ^c^	62.8 ± 18 ^k^	5 ± 6 ^f^	917 ± 115 ^a^

LA: leaf area per leaf; R: roughness of leaf surface; RBA and RBL: ravine breadth of above and lower epidermis, respectively; SD: stomata density of lower epidermis; SL: stomata length; SB: stomata breadth; GC: guard cell area; TN: trichome density of lower epidermis; Tl: trichome length of lower epidermis. Treatments with the same letter were not significantly different.

**Table 4:** Partial regression coefficients (B) and standardized regression coefficients (beta) from a multiple regression analysis of the leaf surface-trait effects on PM accumulation.

Item	B	beta	*p*
Constant	1.533		0.034
LA	0.019	0.445	0.044
R	0.343	0.809	0.012
RBA	–0.007	-0.032	0.749
RBL	0.046	0.125	0.522
SD	–0.010	–0.434	0.041
TL	–0.089	0.052	0.036
SB	–0.135	–0.287	0.357
GC	0.002	0.773	0.125
TN	–0.006	–0.301	0.250
TL	0.0005	0.58	0.628
*R^2^*	0.992		

LA: leaf area per leaf; R: roughness of leaf surface; RBA and RBL: ravine breadth of above and lower epidermis, respectively; SD: stomata density of lower epidermis; SL: stomata length; SB: stomata breadth; GC: guard cell area; TN: trichome density of lower epidermis; Tl: trichome length of lower epidermis.
